# Muddy Water Pneumonitis: An Emerging Occupational Lung Disease in Professional Divers

**DOI:** 10.1002/rcr2.70697

**Published:** 2026-07-29

**Authors:** Sadia Sultana Resma, Dewan Azmal Hussain, Rashedul Hasan Ripon, Fazle Rabbi

**Affiliations:** ^1^ Department of Respiratory Medicine National Institute of Diseases of the Chest and Hospital Dhaka Bangladesh

**Keywords:** environmental exposure, necrotizing pneumonitis, occupational lung disease, professional diving, structural health inequity

## Abstract

Chronic occupational exposure to contaminated aquatic environments is an underrecognized cause of progressive lung injury. Recurrent aspiration of sediment‐rich river water can lead to chronic inflammatory lung injury with possible environmental pathogen colonization and destructive lung disease, even in immunocompetent individuals. A 35‐year‐old non‐smoking professional diver in a tropical mangrove region of the Sundarban, Bangladesh, with 17 years of exposure performing 10–15 daily dives in muddy saline mangrove water, presented with 1 year of progressive exertional dyspnea and intermittent cough. Water aspiration during mud collection was frequent; four coworkers reported similar symptoms. SpO_2_ was 93% at rest, dropping to 89% after 700 m on a 6‐min walk test, indicating exercise‐induced desaturation despite preserved walking distance. Digital clubbing and bilateral coarse crepitations were present. HRCT showed diffuse nodules and a left upper lobe cavitary lesion; Mantoux and Interferon gamma release assay were negative. Pulmonary function revealed severe restrictive defect (TLC 39%, FVC 25%, DLCO 36%, RV/TLC 48%). BAL grew *Acinetobacter* sensitive to meropenem and amikacin. After 7 days of IV therapy, symptoms improved, but domiciliary oxygen was not feasible. The patient died 1 month later. Muddy water pneumonitis can cause progressive necrotizing lung injury in divers, likely representing a multifactorial process involving environmental particulate exposure with superimposed infection. It was worsened by socioeconomic constraints and highlights the need for occupational protection and early recognition.

## Graphical abstract

Chronic exposure to sediment‐rich river water in a professional diver led to progressive necrotizing lung injury mimicking tuberculosis. Bronchoalveolar lavage revealed *Acinetobacter*, highlighting a rare occupational cause of cavitary pneumonitis. This case underscores the impact of environmental exposure and limited access to supportive care on patient outcomes.

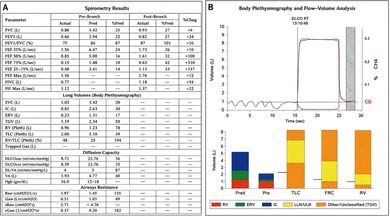

## 1 Introduction

Occupational lung diseases remain a major global health concern, particularly in low‐ and middle‐income countries where protective regulations and surveillance systems are limited. Water‐based occupations involving repeated submersion in sediment‐rich environments may expose workers to chronic microaspiration of contaminated material, potentially predisposing them to inflammatory and infectious pulmonary injury [1, 2].

*Acinetobacter* species are aerobic Gram‐negative coccobacilli widely distributed in soil and water. Although classically associated with healthcare‐acquired infections, they may cause community‐acquired infections in tropical and subtropical regions [1]. Cavitary or necrotizing disease in immunocompetent individuals remains uncommon.

We describe a fatal case of probable occupational muddy water–associated necrotizing pneumonitis in a professional diver from rural Bangladesh, highlighting occupational exposure, radiologic mimicry of tuberculosis and the impact of structural healthcare inequities (Figure 1).

**FIGURE 1:**
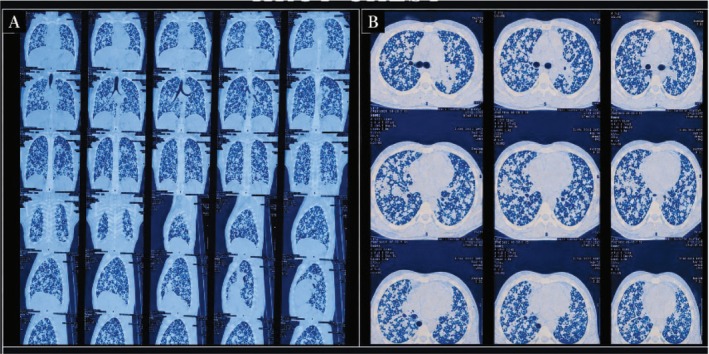
High‐resolution computed tomography (HRCT) findings: (A) Coronal HRCT images demonstrate bilateral, diffuse, discrete, and conglomerated soft‐tissue nodules distributed throughout all lung segments. (B) Enlarged axial HRCT images of the left upper lobe demonstrate an irregular cavitary lesion containing a conglomerated nodule, suggestive of chronic necrotizing lung injury. HRCT, high‐resolution computed tomography.

## 2 Case Report

A 35‐year‐old male professional diver presented with 1 year of progressive exertional dyspnea, intermittent cough, occasional low‐grade fever and 3–4 kg weight loss. There was no hemoptysis, night sweats, or prior chronic illness. He was immunoc negative, a lifelong non‐smoker and had no history of tuberculosis or asthma.

The patient had a 17‐year history as a professional diver in a tropical mangrove region of the Sundarban, Bangladesh. His work involved 10–15 daily dives into muddy saline water, each lasting over 1 min, with frequent involuntary water aspiration. He did not use respiratory protection and routinely collected river sediment manually with his hands and mouth for brick production. Four coworkers reported similar exertional dyspnea, though none had undergone formal medical evaluation.

On physical examination, the patient had a body mass index of 20.4 kg/m^2^. Resting oxygen saturation was 93% on room air, which dropped to 89% following a 6‐min walk distance of 700 m, indicating exercise‐induced desaturation and impaired gas exchange despite preserved functional capacity. Bilateral coarse inspiratory crepitations were audible on lung auscultation, and digital clubbing was present, consistent with chronic hypoxemia and structural lung disease.

Laboratory investigations revealed neutrophilic leukocytosis (Table 1). Sputum testing for tuberculosis, including two GeneXpert assays, was negative, and both the Mantoux test and interferon‐gamma release assay were also negative, effectively excluding latent or active tuberculosis. HIV serology was negative. Bronchoscopy showed a normal bronchial tree and collected bronchoalveolar lavage culture grew *Acinetobacter* species sensitive to meropenem and amikacin, while fungal cultures were negative. These findings supported a diagnosis of community‐acquired bacterial pneumonitis in the context of chronic occupational exposure. However, colonization in the context of structurally abnormal lungs cannot be excluded, particularly given the chronic disease course.

**TABLE 1:** Investigation profile of the patient.

Investigation	Findings	Interpretation
Complete blood count	Haemoglobin 10.9 g/dL white blood cell count 15.6 × 109/L with neutrophil predominance (88%)	Neutrophilic leukocytosis consistent with ongoing inflammatory or infectious process
HIV serology	Negative	No evidence of HIV infection
Sputum GeneXpert MTB/RIF	Negative	No microbiological evidence of pulmonary tuberculosis
Sputum bacterial culture	No growth	No pathogenic bacteria isolated from sputum
Mantoux test	Induration 3 mm	Negative tuberculin skin test
Interferon‐gamma release assay	Negative	Latent or active tuberculosis unlikely
Six‐minute walk test	Oxygen saturation decreased from 93% at rest to 89% after exercise	Exercise‐induced oxygen desaturation
High‐resolution computed tomography (HRCT) of the chest	Bilateral diffuse discrete and conglomerated soft‐tissue nodules involving all lung segments with an irregular cavitary lesion in the left upper lobe	Imaging differential included miliary tuberculosis, metastatic malignancy, and necrotizing infection
Bronchoalveolar lavage	BAL bacterial culture grew *Acinetobacter* spp. sensitive to meropenem and amikacin fungal culture negative GeneXpert MTB/RIF negative NTM multiplex PCR negative	Findings supported bacterial infection without evidence of mycobacterial or fungal disease
Complete pulmonary function test with diffusing capacity (DLCO)	FVC 25% predicted FEV1 22% predicted TLC 39% predicted DLCO 36% predicted RV/TLC 48%	Severe restrictive ventilatory defect with markedly reduced DLCO and elevated RV/TLC suggesting concomitant air trapping.
Electrocardiography	Sinus tachycardia	No significant cardiac abnormality detected
Transthoracic echocardiography	Normal	No significant structural cardiac abnormality detected
Ultrasonography of whole abdomen	Normal	Unremarkable
Serum total IgE	65 IU/mL	Within normal range
C‐ANCA and p‐ANCA	Negative	No serological evidence of ANCA‐associated vasculitis
CT‐guided core biopsy of the left upper lobe cavitary lesion	Chronic fibro‐inflammatory changes with focal necrosis and neutrophilic infiltrates	Histopathological findings consistent with chronic necrotizing pneumonitis

Investigation	Findings	Clinical significance
Lung biopsy		Excluded malignancy and supported chronic necrotizing pneumonitis

*Note:* Summary of laboratory, microbiological, radiological, physiological, cardiac and histopathological investigations performed during the diagnostic evaluation. The findings collectively excluded tuberculosis, non‐tuberculous mycobacterial disease, fungal infection, vasculitis and malignancy, while supporting chronic necrotizing pneumonitis in the setting of prolonged occupational muddy‐water exposure.

Pulmonary function test demonstrated a severe restrictive ventilatory defect with associated diffusion impairment and air trapping (Figure 2). The forced vital capacity (FVC) was 25% of predicted, and the forced expiratory volume in 1 s (FEV_1_) was 22% of predicted. Total lung capacity (TLC) was 39% of predicted, diffusion capacity for carbon monoxide (DLCO) was 36% of predicted, and the residual volume to total lung capacity ratio (RV/TLC) was elevated at 48%, indicating significant air trapping. Lung Biopsy from left cavitary lesion showed chronic fibro‐inflammatory changes with focal necrosis and neutrophilic infiltrates, consistent with chronic necrotizing pneumonitis.

**FIGURE 2:**
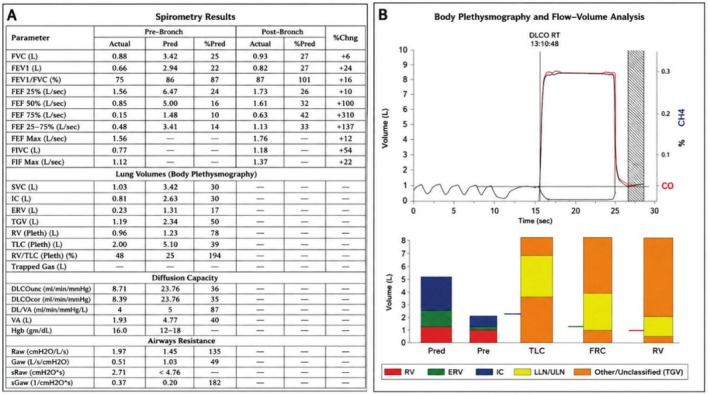
Pulmonary function testing results. (A) Spirometry parameters before and after bronchodilator administration demonstrated severe airflow limitation with significant bronchodilator responsiveness in FEV_1_ (+24%) and mid‐expiratory flow rates. Body plethysmography revealed air trapping with an elevated RV/TLC ratio (48%). Diffusing capacity for carbon monoxide (DLCO) was markedly reduced (35%–36% predicted). Airway resistance was increased with reduced conductance. (B) Representative body plethysmography and single‐breath DLCO analysis. The upper panel shows the DLCO manoeuvre with tracer gas (CH_4_) and carbon monoxide (CO) washout curves. The lower panel illustrates lung volume subdivisions measured by body plethysmography. Pre‐bronchodilator measured values are indicated by the horizontal reference lines relative to the predicted value (Pred) and lower limit of normal (LLN). DL/VA (KCO), diffusing capacity normalized for alveolar volume; DLCO, diffusing capacity of the lung for carbon monoxide; DLCOc, hemoglobin‐corrected diffusing capacity for carbon monoxide; ERV, expiratory reserve volume; FEF, forced expiratory flow; FEV_1_, forced expiratory volume in 1’s; FEV_1_/FVC, ratio of forced expiratory volume in 1’s to forced vital capacity; FIF, forced inspiratory flow; FVC, forced vital capacity; Gaw, airway conductance; IC, inspiratory capacity; Raw, airway resistance; RV, residual volume; RV/TLC, residual volume‐to‐total lung capacity ratio; sGaw, specific airway conductance; sRaw, specific airway resistance; SVC, slow vital capacity; TGV, thoracic gas volume; TLC, total lung capacity; VA, alveolar volume.

The patient received intravenous meropenem and amikacin for 7 days, with short‐term improvement in oxygen saturation and exercise tolerance. Although *Acinetobacter* species can represent colonization, in this patient the combination of progressive symptoms, diffuse nodular and cavitary lesions on HRCT, and short‐term improvement after targeted intravenous antibiotics suggests a possible contributory role, but does not establish definitive causality.

He was discharged with medical advice for continuous domiciliary oxygen support. However, prolonged therapy and continuous oxygen supplementation were not feasible due to severe financial constraints, lack of home oxygen availability nearby, and unreliable electricity supply in his remote village to run the oxygen concentrator. One month later, he died at home, reportedly from progressive respiratory failure without sustained oxygen support.

## 3 Discussion

Occupational inhalational exposures are recognized as important contributors to progressive lung disease beyond classical pneumoconioses [3]. Chronic exposure to organic particulates and environmental contaminants has been associated with interstitial and fibrotic lung injury, particularly where protective measures are absent. Water‐related lung injury has previously been described following aspiration events, near‐drowning and occupational exposures, while chronic repetitive exposure remains underreported [1].

In this patient, 17 years of daily diving in sediment‐rich river water likely led to repeated microaspiration, which may have contributed to chronic airway inflammation and persistent colonization by environmental pathogens. Over time, this may have contributed to progressive parenchymal injury, characterized by cavitary necrosis and structural lung damage. The combination of severe restrictive physiology, markedly reduced DLCO, air trapping and digital clubbing suggests chronic structural remodelling rather than isolated acute infection.

*Acinetobacter* species are well recognized environmental organisms, usually survive in aquatic, sediment‐rich habitats. Although traditionally considered nosocomial pathogens, community‐acquired *Acinetobacter* pneumonia has been increasingly reported in tropical regions, often presenting as severe or necrotizing disease [4]. Chronic exposure may predispose to lower respiratory tract colonization. Cavitary manifestations are uncommon in immunocompetent individuals and may reflect prolonged inflammatory injury or delayed treatment.

Chronic exposure to sediment‐rich environments also raises the possibility that desiccated sludge particles may become aerosolized and inhaled, potentially producing a pneumoconiosis‐like process. However, the radiological findings in this case are not typical of classical pneumoconiosis, and the absence of high‐magnification histopathological analysis limits definitive characterization.

Radiologically, the presence of diffuse nodular infiltrates with cavitation necessitated exclusion of tuberculosis and malignancy. Tuberculosis was effectively excluded through repeated negative molecular and immunologic investigations. Although malignancy was considered based on imaging findings, the absence of supportive histopathological evidence and the lack of progressive features suggestive of neoplasia made this diagnosis unlikely. In contrast, the microbiologic isolation of *Acinetobacter* from bronchoalveolar lavage, together with short‐term clinical response to targeted antimicrobial therapy, supported a diagnosis of infectious pneumonitis. However, its detection in bronchoalveolar lavage does not necessarily indicate primary causation, particularly in patients with underlying structural lung disease. In this case, *Acinetobacter* may represent either a true pathogen or a secondary colonizer, and its precise contribution to the observed lung injury remains uncertain.

Over time, repeated microaspiration may result in progression from initial airway inflammation to deeper parenchymal injury, fibrotic remodelling and cavitary necrosis. Notwithstanding the limitations, the convergence of long‐term occupational exposure, compatible radiologic findings, microbiologic evidence, and therapeutic response suggests a possible diagnosis of exposure‐related pneumonitis with superimposed infection (Figure 3); however, definitive attribution to a single aetiology is not possible.

**FIGURE 3:**
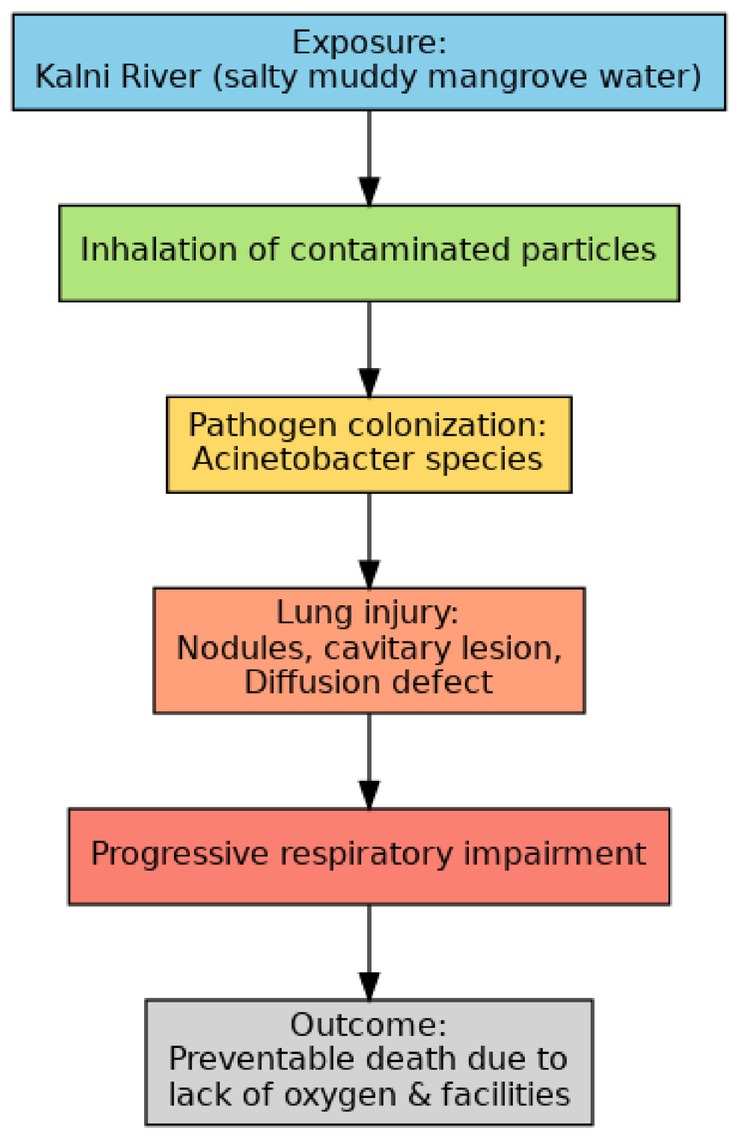
Flow chart of the pathophysiology of development of muddy water pneumonitis.

This case has several limitations. The clinical presentation was non‐specific and overlapped with tuberculosis, interstitial lung disease, and other occupational lung disorders, making diagnosis challenging. The absence of standardized diagnostic criteria necessitated reliance on exposure history and exclusion of alternative diagnoses.

Notably, despite a relatively preserved 6‐min walk distance (700 m), the patient demonstrated exercise‐induced desaturation (SpO_2_ 93%–89%), suggesting impaired gas exchange. This may reflect a clinical–functional mismatch, where preserved exercise capacity—potentially related to occupational physical conditioning—coexists with underlying parenchymal lung disease. Importantly, exercise‐induced desaturation during the 6‐min walk test is considered a more sensitive indicator of gas exchange abnormality than walking distance alone [5, 6].

Chronic water exposure also raises the possibility of multiple infectious etiologies, including bacterial, fungal, and atypical mycobacterial pathogens. Microbiological evidence was limited to a single bronchoalveolar lavage isolate, which may not definitively distinguish infection from colonization. In addition, histopathological evaluation was based on a single‐site biopsy demonstrating non‐specific chronic inflammatory changes; lack of high‐magnification analysis further limits definitive diagnosis, and sampling error cannot be excluded. No established treatment protocols exist, and management remains largely supportive, with risk of progressive and irreversible lung damage. Such cases may be underrecognized or misdiagnosed as tuberculosis or hypersensitivity pneumonitis, limiting accurate epidemiological characterization. Besides, the disease course extending over one year is more consistent with chronic inflammatory process from occupational lung injury rather than an isolated acute infectious event. Muddy water pneumonitis may represent a potential occupational lung disease in divers. This case highlights a likely multifactorial aetiology, involving environmental particulate exposure and possible secondary infection, with outcomes potentially worsened by socioeconomic constraints.

## Author Contributions

Sadia Sultana Resma played the central role in this study, being closely involved in the patient's clinical care, developing the concept of the report, conducting the literature review, and taking the lead in drafting and refining the manuscript. She coordinated the overall work and integrated inputs from all co‐authors into the final version. Dewan Azmal Hussain provided senior oversight throughout the process, guided the clinical approach and interpretation, and critically reviewed the manuscript to strengthen its intellectual content. Rashedul Hasan Ripon contributed to the literature review, data organization, and assisted in shaping the manuscript. Fazle Rabbi supported data collection, literature search, and provided assistance during manuscript preparation. All authors have reviewed and approved the final manuscript and agree to be accountable for the integrity and accuracy of the work.

## Funding

The authors have nothing to report.

## Consent

The authors declare that written informed consent was obtained for the publication of this manuscript and accompanying images and attest that the form used to obtain consent from the patient(s) complies with the Journal requirements as outlined in the author guidelines.

## Conflicts of Interest

The authors declare no conflicts of interest.

## Data Availability

The data that support the findings of this study are available on request from the corresponding author. The data are not publicly available due to privacy or ethical restrictions.
